# Osteogenic Differentiation Potential of iMSCs on GelMA-BG-MWCNT Nanocomposite Hydrogels

**DOI:** 10.3390/biomimetics9060338

**Published:** 2024-06-03

**Authors:** Rebeca Arambula-Maldonado, Kibret Mequanint

**Affiliations:** 1 School of Biomedical Engineering, The University of Western Ontario, 1151 Richmond Street, London, ON N6A 5B9, Canada; rarambul@uwo.ca; 2 Department of Chemical and Biochemical Engineering, The University of Western Ontario, 1151 Richmond Street, London, ON N6A 5B9, Canada

**Keywords:** osteogenic differentiation, electrically conductive, nanocomposite hydrogels, cell adhesion, mineralization

## Abstract

The ability of bone biomaterials to promote osteogenic differentiation is crucial for the repair and regeneration of osseous tissue. The development of a temporary bone substitute is of major importance in enhancing the growth and differentiation of human-derived stem cells into an osteogenic lineage. In this study, nanocomposite hydrogels composed of gelatin methacryloyl (GelMA), bioactive glass (BG), and multiwall carbon nanotubes (MWCNT) were developed to create a bone biomaterial that mimics the structural and electrically conductive nature of bone that can promote the differentiation of human-derived stem cells. GelMA-BG-MWCNT nanocomposite hydrogels supported mesenchymal stem cells derived from human induced pluripotent stem cells, hereinafter named iMSCs. Cell adhesion was improved upon coating nanocomposite hydrogels with fibronectin and was further enhanced when seeding pre-differentiated iMSCs. Osteogenic differentiation and mature mineralization were promoted in GelMA-BG-MWCNT nanocomposite hydrogels and were most evidently observed in the 70-30-2 hydrogels, which could be due to the stiff topography characteristic from the addition of MWCNT. Overall, the results of this study showed that GelMA-BG-MWCNT nanocomposite hydrogels coated with fibronectin possessed a favorable environment in which pre-differentiated iMSCs could better attach, proliferate, and further mature into an osteogenic lineage, which was crucial for the repair and regeneration of bone.

## 1. Introduction

The development of an electrically conductive composite is proposed as a promising biomaterial for bone tissue engineering. Natural bone is a complex organic–inorganic composite tissue composed of collagen–hydroxycarbonate apatite that possesses endogenous electrically conductive properties in response to mechanical forces [1,2,3]. Composite material approaches offer a solution to mimicking the various physiological properties possessed by complex tissue systems [1,4]. Since Fukada and Yasuda discovered the electrically conductive properties of bone [2], the development of electrically conductive materials has been emerging for bone tissue engineering to mimic bone’s natural structural and electrically conductive nature. The greatest attention, however, has been on the incorporation of an electrically conductive component in bone biomaterials that can deliver electrical cues through the application of electrical stimulation for the maturation of osteoblasts and the promotion of the repair and regeneration of bone defects [5,6,7].

The design and fabrication of any bone biomaterial should entail several requirements. Successful bone reconstruction requires osteoproduction, osteoinduction, osteoconduction, and vascularization [6,8]. Bone biomaterials undergo different processes in which they can be implanted for clinical translation. In the first approach, cells are seeded to the biomaterial and are matured in a bioreactor with constant media flow and mechanical stimulation conditions [9]. In the second method, the bone biomaterials, with or without seeded cells, are implanted into the bone defect, in which the patient’s body acts as a natural bioreactor to promote the regeneration of tissue [9]. In both approaches, the bone biomaterial serves as a temporary bone substitute in which the cells lay their extracellular matrix (ECM) on the surface of the implant, which is eventually resorbed and replaced over time in tune with the newly regenerated tissue [1,9].

Biodegradable nanocomposite hydrogels composed of gelatin methacryloyl (GelMA), sol–gel-derived tertiary bioactive glass (BG), and uniformly dispersed multiwall carbon nanotubes (MWCNT) have been previously developed in an attempt to mimic the organic–inorganic structural composition of bone with tunable electrical and electro-mechanical properties [1]. GelMA is derived from gelatin, which is the hydrolyzed form of type I collagen found in bone. The application of GelMA as a polymer for developing a nanocomposite is, therefore, ideal for creating an environment that mimics the main organic component of endogenous bone. Its main advantages include stability and possessing a slower degradation rate compared to gelatin while conferring toughness to the inorganic component. In addition, GelMA possesses an arginine-glycine-aspartic acid (RGD) sequence that could further favor the cell-ECM interactions, thus promoting cell adhesion [1,10].

Furthermore, BGs have long been studied for their osteoconductive and osteoinductive potential stemming from the release of stimulatory ions, which promote bonding to the bone [11,12]. Calcium phosphates (CaP), specifically hydroxyapatite (HA) (Ca_10_(PO_4_)_6_(OH)_2_) and β-tricalcium phosphate (β-TCP) (Ca_3_(PO_4_)_2_), are alternative inorganic components used in bone biomaterials due to their osteoconductive and bioresorbable properties [13,14,15]. HA is the most crystalline ceramic and is, therefore, very stable when implanted into damaged bone sites, possessing a very slow rate of reabsorption, which is not in tune with the formation of new bone tissue [16]. In contrast, β-TCP is a less crystalline ceramic that reabsorbs more rapidly in the human body [17]. As β-TCP degrades, there is an increased concentration of calcium and phosphate on the surface of the ceramic, which enhances the mineralization and formation of bone. However, the mechanical strength of β-TCP is lower due to the rapid resorption in the body, compromising the temporary mechanical support of the repairing bone at the site of implantation. The high crystalline form of β-TCP can be achieved by sintering to obtain a mechanically stronger ceramic; however, the reabsorption rate would become very slow, preventing bone repair. For these reasons, the application of sol–gel-derived BGs as an inorganic component to develop bone biomaterials has been of great interest due to their biocompatibility, osteoconductivity, biodegradability, and ability to form bone-mimetic mineral phases at their interfaces in physiological conditions and in tune with the newly regenerated tissue [18].

The development of the original melt-quenched BG (46.1%, SiO_2_, 24.4%, Na_2_O, 26.9% CaO, and 2.6% P_2_O_5_, in mol%), 45S5 Bioglass^®^, by Hench and his colleagues [19], has made possible the formulation of various BGs, including sol–gel-derived glasses, and investigate their bioactive behavior in vitro and in vivo [20,21,22]. Melt-quenched BGs consist of melting the oxide components at elevated temperatures (~1300 °C), followed by quenching the molten liquid to form a glass [19,23]. Sol–gel-derived BGs, however, are produced through the hydrolysis and polycondensation of alkoxide precursors, forming a gel, which is subsequently aged and dried [24]. The sol–gel method is preferred over the melt-quenching technique since it offers several advantages. Some advantages include the low processing temperatures required to produce BGs, which enable the addition of a polymer to create organic–inorganic bone biomaterials. In addition, sol–gel-derived BGs are more porous, resulting in a higher surface area and, thus, enhancing their bioactivity. The bioactivity of BG occurs due to the reabsorption of the glass, resulting in the formation of a hydroxycarbonate apatite (HCA) layer on its surface and bondage to bone [19,23]. Bioactivity of sol–gel-derived BGs can be achieved with compositions of 90 mol% silica, whereas osteoconduction and osteoinduction property is achieved at a silica content of 60 mol% or less in melt-derived BGs [25]. Therefore, a sol–gel-derived tertiary BG composition consisting of 70 mol% silica could be a suitable formulation for the development of bioactive organic–inorganic bone biomaterials.

Finally, the incorporation of MWNCT into GelMA-BG nanocomposites is proposed as a promising bone biomaterial that can support cellular bioactivity, act as a reinforcement element, mimic the electrical properties of native bone, and promote the formation and maturation of bone through the application of exogenous electrical stimulation. Carbon-based conductive materials, such as MWCNT, have the advantage that they can be synthesized in various geometrical and morphological structures that can alter their physiological responses and, hence, their ability to regenerate bone defects [1]. Therefore, we were interested in incorporating MWCNT into organic–inorganic nanocomposites to assess their functions as potential bone biomaterials. Furthermore, evaluations of the osteogenic ability of potential bone biomaterials should be performed in vitro and preferably in human-derived cells since they could better resemble clinically driven outcomes. In vitro evaluations could be performed in either bone cells or stem cells, such as mesenchymal stem cells (MSCs). MSCs are multipotent and have the ability to differentiate into osteoblasts, chondrocytes, and adipocytes [26]. However, the availability of MSCs is restricted and related to complications, including donor site comorbidity associated with the invasive isolation from bone marrow or other tissues, such as fat [27,28]. In addition, the differentiation and proliferation capacity of MSCs decrease with donor age and the duration of culture [29]. Therefore, MSCs derived from human induced pluripotent stem cells (iPSCs), termed iMSCs, represent an alternative to primary MSCs and, in addition, present several advantages. Some advantages include their generation from well-characterized and banked iPSCs with known human leukocyte antigen type [27] and their successful application in vivo to aid bone regeneration by their direct osteogenic differentiation and their recruitment of host cells in mice models [28].

Herein, GelMA-BG-MWCNT nanocomposite hydrogels were evaluated to assess their viability with iMSCs. Since iMSCs are human-derived, their in vitro applications with GelMA-BG-MWCNT nanocomposite hydrogels could better resemble clinical translational outcomes. Good cytocompatibility properties were observed on nanocomposite hydrogels, presenting an increased initial cell adhesion of pre-differentiated cells after coating biomaterials with fibronectin, which was a crucial step to further evaluate their osteogenic ability and matrix mineralization. GelMA-BG-MWCNT nanocomposite hydrogels could become promising biomaterials for the repair and regeneration of bone that could potentially be applied for in vivo studies and further applications in clinical translations.

## 2. Materials and Methods

### 2.1. Materials

Gelatin type A (porcine skin) (G2500, gel strength (Bloom No.) 300 with viscosity average molecular weight of 100 kDa), methacrylic anhydride (containing 2000 ppm topanol A as an inhibitor, 94%), potassium persulfate, diacrylated pluronic F-127, multiwall carbon nanotube (MWCNT, >98% carbon basis, O.D. × L 6–13 nm × 2.5–20 μm), tetraethyl orthosilicate (TEOS, 98%), triethyl phosphate (TEP, 99.8%), and Alizarin Red S were purchased from Sigma-Aldrich (Milwaukee, WI, USA). Calcium ethoxide was obtained from Gelest Inc. (Morrisville, PA, USA). N,N,N′,N′-tetramethylethane-1,2-diamine (TEMED) was purchased from Merck KGaA (Darmstadt, Germany). Dulbecco’s Modified Eagle’s Medium (DMEM), Hanks’ Balanced Salt Solution (HBSS), Fetal Bovine Serum (FBS), penicillin/streptomycin (pen/strep), and live/dead cell imaging kit were acquired from Thermo Fisher (Waltham, MA, USA). Alexa Fluor^®^ 488 phalloidin and 4′6-diamidino-2-phenylindole (DAPI) were purchased from Life Technologies (Burlington, ON, Canada).

### 2.2. Synthesis of Gelatin Methacryloyl (GelMA)

Gelatin type A (porcine skin) was mixed at 10% *w*/*v* in phosphate-buffered saline (PBS) at 40 °C until fully dissolved. A 10% *v*/*v* of methacrylic anhydride was added dropwise under stirring to the viscous gelatin solution and allowed to react for 1 h at 40 °C. To stop the reaction, a 5× dilution of warm PBS was added and dialyzed against distilled water using a 12–14 kDa cutoff dialysis tube for one week to remove unreacted components. The solution was vacuum dried at 40 °C, and GelMA prepolymer was stored at 4 °C until use [1]. 

### 2.3. Synthesis of Tertiary Bioactive Glass (BG)

BG was prepared by a sol–gel process which consisted of hydrolyzing TEOS and TEP together with a catalytic amount of 1M HCl under vigorous stirring at room temperature (RT). Calcium ethoxide was dissolved separately in 2-ethoxyethanol and was added dropwise to the hydrolyzed TEOS until a gel was formed. The BG was aged for two days at RT, followed by drying under vacuum at 50 °C to obtain a final molar composition of 70% SiO_2_, 26% CaO, and 4% P_2_O_5_. The final product was ground to a fine powder and stored at RT until further use [1,30].

### 2.4. Preparation of GelMA-BG-MWCNT Nanocomposite Hydrogel Biomaterials

Diacrylated pluronic F-127 surfactant was used to prepare MWCNT stock dispersions. The surfactant was dissolved in water at high temperature to a concentration of 20 mg/mL, followed by adding 20 mg/mL MWCNT. The dispersion was sonicated for 1 h at 50 °C and stored at RT until further use for GelMA-BG-MWCNT biomaterial preparation. GelMA prepolymer was dissolved in water at a concentration of 10% *w*/*v*. 1 and 2 wt.% MWCNT were added to the GelMA prepolymer solution, followed by sonication at 50 °C for 30 min. BG powder was subsequently added to GelMA-MWCNT mix at different concentrations and was further sonicated for 30 min. KPS and TEMED were added separately as the thermal initiator and accelerator, respectively, at concentrations of 0.5% *w*/*v* with respect to the total prepolymer concentration to crosslink GelMA at 60 °C. Sample nomenclature is presented in Table 1.

### 2.5. Cytotoxicity of iMSCs Cultured on GelMA-BG-MWCNT Nanocomposite Hydrogels

Mesenchymal stem cells derived from human induced pluripotent stem cells, hereinafter named iMSCs (kindly donated by Dr. Dale Laird, Western University, London, ON, Canada), were used for in vitro cell culture studies. The primary cell source used to generate iPSCs was human dermal fibroblasts [31]. Reprogramming of human dermal fibroblasts into iPSCs was performed by the Centre for the Commercialization of Regenerative Medicine (CCRM, Toronto, ON, Canada) [31]. Briefly, dermal fibroblasts were transduced with the Yamanaka factors (hOct4, hSox2, hKlf4, and hc-Myc) using the non-integrating Sendai virus [31,32]. After seven days of induction, transduced cells were enzymatically dissociated and plated onto Matrigel-coated (Corning, NY, USA; #354277) 10 cm dishes and fed daily with mTeSR1 (StemCell Technologies, Vancouver, BC, Canada; #07174) until approximately day 28, when presumptive iPSC colonies were ready [4]. Individual colonies were picked into individual feeder-free 6-well plate-coated dishes. Characterizations of iPSCs were performed to ensure successful reprogramming [31]. Furthermore, iPSCs were differentiated into MSCs, thus, iMSCs, using the STEMdiff mesenchymal progenitor kit (StemCell Technologies #05240, Vancouver, BC, Canada) according to the manufacturer’s instructions [33]. Appropriate characterizations were performed to ensure iMSC differentiation [33]. Tissue culture plates (TCP) used for iMSCs maintenance were coated with gelatin diluted to 0.1% *w*/*v* in PBS and incubated at 37 °C for 1 h before aspirating gelatin solution and plating iMSCs. Cells were cultured in mesenchymal stem cell expansion media (MSCEM, Cedarlane Labs, Burlington, ON, Canada; HMSC.E.MEDIA-450) supplemented with 10% FBS and 1% pen/strep. Nanocomposite hydrogels were disinfected under ultraviolet (UV) light for 30 min and pretreated in HBSS, followed by coating in 0.1% *w*/*v* gelatin. iMSCs were seeded directly onto biomaterials at a density of approximately 15,625 cells/cm^2^. A live/dead cell staining kit was used to detect the viability of cells after 1, 3, and 7 days of culture and was used according to the manufacturer’s protocol. Tissue culture plate (TCP) was used as control. Images were taken with a Leica DMi8 fluorescence microscope (Leica Microsystems CMS GmbH, Wetzlar, Germany). All in vitro experiments were conducted in triplicate. Unless specified, all experiments were carried out in a 24-well plate with nanocomposite hydrogel dimensions of 10 mm diameter and 5 mm height.

### 2.6. Adhesion of Cells on Gelatin- and Fibronectin-Coated GelMA-BG-MWCNT Nanocomposite Hydrogels

Nanocomposite hydrogels were disinfected under ultraviolet (UV) light for 30 min and pretreated in HBSS, followed by coating in either 0.1% *w*/*v* gelatin or 5 μg/mL fibronectin and incubated at 37 °C for 1 h. iMSCs were subsequently seeded directly onto coated biomaterials at a density of approximately 15,625 cells/cm^2^. Alternatively, iMSCs were pre-differentiated using osteogenic induction media (100 nM dexamethasone, 50 μg/mL L-ascorbic acid, and 10 mM Na_x_H_3−x_PO_4_) for 5 days and then seeded at a density of ~15,625 cells/cm^2^ onto fibronectin-coated nanocomposite hydrogels. After 1 day of culture, cells were fixed using 4% paraformaldehyde (EMD Chemicals Inc. Gibbstown, NJ, USA) and were permeabilized for 10 min with 0.5% Triton X-100 in PBS, followed by blocking cells with 1% BSA in PBS for 2 h at RT. Primary antibody incubation with anti-vinculin (1:100; MAB3574, clone VIIF9, EMD Millipore) was incubated overnight at 4 °C. After washing three times with PBS, Alexa Fluor 594 goat anti-mouse IgG secondary antibody (1:300; Thermo Fisher, Burlington, ON, Canada) was used to detect primary antibody binding. Cell cytoskeleton was stained with Alexa Fluor^®^ 488 conjugated phalloidin (1:100) and counterstained with DAPI (300 nmol in PBS) to visualize cell nuclei.

### 2.7. Osteogenic Gene Expression of Differentiated iMSCs Cultured on GelMA-BG-MWCNT Nanocomposite Hydrogels

Nanocomposite hydrogels were disinfected under UV light for 30 min and pretreated in HBSS. iMSCs cultured in gelatin-coated dishes were differentiated for 5 days using osteogenic induction media (100 nM dexamethasone, 50 μg/mL L-ascorbic acid, and 10 mM Na_x_H_3−x_PO_4_). Pre-differentiated cells were seeded at a density of ~15,625 cells/cm^2^ onto fibronectin-coated nanocomposite hydrogels. After 15 days of further differentiation, total RNA was extracted from cells using the Bio-Rad Aurum™ Total RNA Mini Kit (Mississauga, ON, Canada) according to the manufacturer’s protocol for osteogenic gene expression experiments. A complementary DNA (cDNA) template was prepared by using 1 μg of total RNA primed with random primers, according to Promega™ Random Hexamers protocol (Thermo Fisher). Quantitative real-time PCR (qRT-PCR) was conducted in 10 μL of reaction volumes using a CFX96™ Real-Time System (C1000 Touch Thermal Cycler; Bio-Rad, Mississauga, ON, Canada) and measured with iQ™ SYBR^®^ Green Supermix (Bio-Rad), according to the recommended procedures. Table 2 presents the sequences of primers used. iMSCs differentiated on pure GelMA (100-0-0) hydrogels were used as a control to assess the effects of BG and MWCNT on the induction of osteogenic differentiation. The results were analyzed with the comparative threshold cycle method, normalized with mouse 18S as an endogenous reference, and reported as relative values (ΔΔ CT) to the control.

### 2.8. Western Blot Analysis of Differentiated iMSCs Cultured on GelMA-BG-MWCNT Nanocomposite Hydrogels

Evaluation of the levels of osteogenic proteins was performed through Western blotting. Pre-differentiated cells were seeded onto disinfected and fibronectin-coated nanocomposite hydrogels. In brief, after further differentiating for 15 days, cells cultured on nanocomposite hydrogels were lysed in buffer containing 150 mM NaCl, 10 mM Tris-HCl (pH 7.4), 1 mM EDTA, 0.5% Nonidet P-40, and 1% Triton X-100 and supplemented with protease inhibitor (Roche Applied Science, Indianapolis, IN, USA). Protein concentrations were determined by Quick Start™ Bradford Protein Assay (Bio-Rad, Mississauga, ON, Canada), and 50 μg of total protein lysate was resolved on 10% SDS-PAGE and subsequently transferred to nitrocellulose membrane. The following primary antibodies were used for immunoblotting: anti-Osteopontin (rabbit, 1:2000; Abcam, ab8448); and anti-Osteocalcin (rabbit, 1:250; Abcam, ab133612). Primary antibody labeling was detected using HRP-conjugated goat anti-rabbit secondary antibody and the ECL detection system.

### 2.9. Immunofluorescence Microscopy

Assessment of osteogenic protein expression was evaluated on pre-differentiated cells that had been further differentiated for 15 days on fibronectin-coated GelMA-BG-MWCNT nanocomposite hydrogels. Cells were fixed using 4% paraformaldehyde (EMD Chemicals Inc. Gibbstown, NJ, USA) and were permeabilized for 10 min with 0.5% Triton X-100 in PBS, followed by blocking cells with 1% BSA in PBS for 3 h at RT. Samples were labeled with the following antibodies overnight at 4 °C: osteopontin (rabbit, 1:100; Abcam, ab8448); osteocalcin (rabbit, 1:100; Abcam, ab133612). Primary antibody binding was detected using Alexa Fluor^®^ 555 goat anti-rabbit IgG. Counterstaining with Alexa Fluor^®^ 488 conjugated phalloidin (1:100) and DAPI (300 nmol in PBS) were used to visualize F-actin and cell nuclei, respectively.

### 2.10. Evaluation of Mineralization of Differentiated iMSCs Cultured on GelMA-BG-MWCNT Nanocomposite Hydrogels

The mineralization ability of pre-differentiated iMSCs co-cultured with disinfected nanocomposite hydrogels was assessed by staining with Alizarin Red solution (pH = 4.2) for 5 min at RT. Glass coverslips were placed in TCP, followed by the addition of nanocomposite hydrogels (6 mm diameter and 2 mm height). After 15 days of further differentiating cells co-cultured with GelMA-BG-MWCNT hydrogels, glass coverslips were dehydrated in acetone and immersed in an acetone-xylene (1:1) solution. Samples were cleared in 95% and 100% ethanol, followed by dipping in a xylene solution. Coverslips were subsequently tipped onto a synthetic mounting media for visualization. For quantification of Alizarin Red staining, pre-differentiated cells were cultured on the nanocomposite hydrogels (10 mm diameter and 5 mm height) and further differentiated for 15 days. After the differentiation period, samples were fixed with 70% ethanol at RT for 15 min, followed by washing with distilled water. An amount of 40 mM of Alizarin Red staining was added and incubated for 30 min at RT. Unincorporated dye was aspirated, followed by washing four times with distilled water under shaking for 5 min each time. A total of 10% *v*/*v* acetic acid was added and incubated for 30 min under shaking at RT. The monolayer was scraped from the nanocomposite hydrogels and transferred to a microcentrifuge tube, followed by vortexing for 30 s. The slurry was overlaid with mineral oil, heated to 85 °C for 10 min, and transferred to ice for 5 min. Samples were centrifuged, and supernatants were transferred to a new tube, followed by the addition of 10% *v*/*v* ammonium hydroxide to neutralize the acid. Samples were aliquoted in triplicate in a 96-well plate, and absorbance was measured at 405 nm [34].

### 2.11. Statistical Analysis

Statistical analysis of the data was performed using GraphPad Prism Version 10.1.2 (324). Differences were tested by one-way ANOVA, and a *p*-value of <0.05 was used for statistical significance.

## 3. Results

### 3.1. Cell Viability of Mesenchymal Stem Cells Derived from Human Induced Pluripotent Stem Cells (iMSCs) on GelMA-BG-MWCNT Nanocomposite Hydrogels

**Figure 1:**
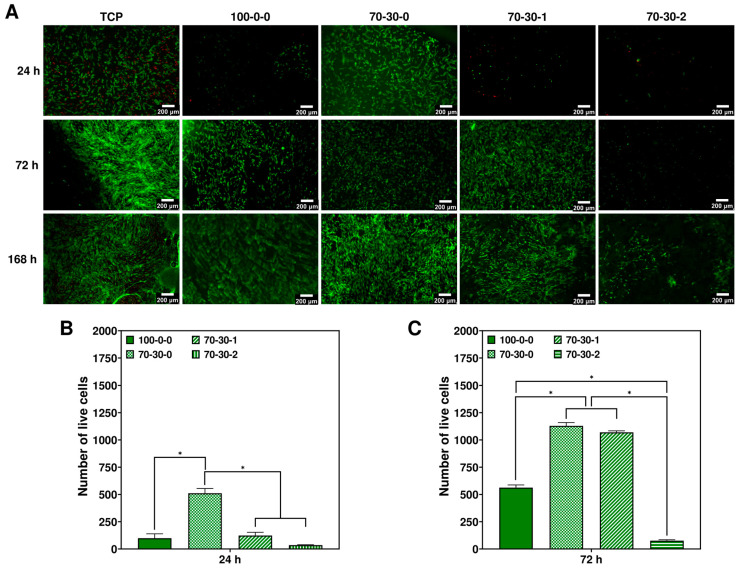
Viability of iMSCs on GelMA-BG-MWCNT nanocomposite hydrogels. (**A**) Live/dead staining of iMSCs cultured on nanocomposite hydrogels after 24, 72, and 168 h. (green = live cells; red = dead cells). Scale bar = 200 μm. Number of live iMSCs at (**B**) 24 h and (**C**) 72 h culture. * *p* < 0.05.

Attachment and proliferation of targeted cells to the biomaterial is a fundamental step in tissue engineering. The digital images of the pure GelMA (100-0-0) hydrogel, 70-30-0, 70-30-1, and 70-30-2 nanocomposite hydrogels used to study iMSCs are shown in Figure S1 (Supporting Information). To assess the viability of cells, the undifferentiated state of iMSCs need to be maintained by coating TCP surfaces with a biomimetic microenvironment in the form of adhesion or extracellular matrix (ECM) proteins for their attachment [35,36,37]. Gelatin was used as a substrate to coat nanocomposite hydrogels prior to seeding iMSCs. Figure 1A depicts the live/dead staining images of iMSCs cultured on nanocomposite hydrogels for 24, 72, and 168 h. After 24 h of culture, pure GelMA (100-0-0) hydrogel and nanocomposite materials containing 1 and 2 wt.% MWCNT presented a significant decreased initial cell attachment (*p* < 0.05) compared to the 70-30-0 hydrogel (Figure 1A,B). The initial cell viability at 24 h culture for the 70-30-0 hydrogel was comparable to that of the TCP control. Evaluation of the adhesion ability of human MSCs (hMSCs) has also been studied on methacrylated hyaluronic acid (MeHA) hydrogels and MeHA hydrogels conjugated with cell-adhesive ligands (RGD)-bearing silica nanoparticles (MeHA-SiO_2_) [38]. The findings of this study showed that hMSCs adhered better to the MeHA-SiO_2_ hydrogels but failed to adhere or spread well on the MeHA hydrogels after only 24 h culture [38]. It also revealed that mature focal adhesion points, cell-ECM adhesion structures, were formed on the MeHA-SiO_2_ hydrogels since the immobilization of SiO_2_ nanoparticles conjugated with RGD was an important nanomaterial to incorporate in a soft polymeric matrix to regulate critical cellular responses [38]. Similar to the results from this study, pure 100-0-0 hydrogels presented a decreased initial cell adhesion compared to the 70-30-0 hydrogels. The BG in the 70-30-0 hydrogels plausibly functions as anchorage points to enable cell attachment and spreading. The viability and proliferation of cells increased at 72 h of culture for all nanocomposite hydrogels, but it was significantly higher for the 70-30-0 and 70-30-1 hydrogels (*p* < 0.05) (Figure 1B). Furthermore, at 168 h of culture, iMSCs continued to grow considerably on the hydrogels with minimal cell death, thus increasing their viability in function of culture time. These results indicate that iMSCs take up to three days to adapt to the 100-0-0, 70-30-1, and 70-30-2 hydrogels, after which cell density and viability continue to increase. Although cell viability increased on the 70-30-2 hydrogel after 72 and at 168 h of culture, the rate at which cells proliferated was slower compared to the rest of the biomaterials and did not show evidence of cytotoxic response due to the addition of 2 wt.% MWCNT. The delayed cell proliferation observed in the 70-30-2 hydrogels was due to the initial low cell adhesion/retention at 24 h, which affected the cell numbers at 168 h of culture [1]. In addition, it takes more effort for iMSCs to attach to higher concentrations of hydrophobic surfaces, such as MWCNT, which was the reason why the initial cell adhesion on the 70-30-2 hydrogels was not as favorable [1]. Moreover, the initial cell attachment at 24 h on the 70-30-1 hydrogel was low, after which the cell density and viability considerably increased until reaching 168 h of culture. Although it has been known that stem cells attach and spread well to a rigid substrate, such as TCP or a glass coverslip [38,39], MWCNT possess a rough topography. It has been shown that the adhesion of hMSCs is regulated by interfacial roughness, in which cell adhesion and spreading decrease as the roughness of surfaces increase [40]. However, after 24 h of culture, the 70-30-1 hydrogel might adsorb proteins due to the highly delocalized π-bonds of the hydrophobic nature of MWCNT [5], thus enhancing the spread of iMSCs on the nanocomposite hydrogel [1].

Differentiation of cells takes place at the expense of proliferation and would normally be initiated at a confluency of around 70%. However, according to the live/dead images, 70% confluency would be reached between days two and three for the cases of all hydrogels (Figure 1A) except for the 70-30-2, which is significantly lower (*p* < 0.05) (Figure 1B,C). In addition, cells growing on the 100-0-0 hydrogel are also low compared to the 70-30-0 and 70-30-1 hydrogels (*p* < 0.05) at 72 h of culture (Figure 1C). Therefore, the assessment of the osteogenic ability of cells cultured on nanocomposite hydrogels could not be evaluated since the initial cell density baseline is not even amongst the biomaterials, limiting the performance of appropriate comparisons.

**Figure 2:**
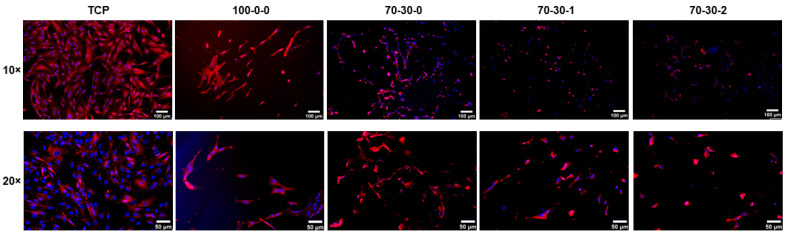
Immunofluorescence microscopy images of iMSCs cultured on gelatin-coated GelMA-BG-MWCNT nanocomposite hydrogels. Focal adhesions of iMSCs cultured on hydrogels (blue = nuclei; red = vinculin) at 24 h of culture. A 10× scale bar = 100 µm. A 20× scale bar = 50 µm.

Understanding the interfacial interactions between iMSCs and nanocomposite hydrogels allows us to determine which culture condition requires modification to improve the initial cell adhesion until reaching an adequate and similar cell density among the biomaterials to induce osteogenic differentiation. Formation of focal adhesions is the initial stage of cell adherence, with the substrates showing effective cell–biomaterial interactions necessary for cell migration, proliferation, and signal transduction [41]. Vinculin is a focal adhesion protein involved in anchoring actin filaments to integrin adhesive molecules [41]. Investigation of the presence of vinculin in cells cultured on nanocomposite hydrogels allows for the evaluation of the degree of integrin-mediated communication between the iMSCs and the nanocomposite hydrogels. iMSCs were stained for vinculin (red) and nuclei (blue) at 24 h of culture to observe the cell–biomaterial interfacial interactions (Figure 2). It is observed that the number of cells attached to the hydrogels is less than that in the control glass coverslip. This is mostly evident for the 100-0-0, 70-30-1, and 70-30-2 hydrogels, which is similar to the results shown in the live/dead staining (Figure 1A). Although the 70-30-0 hydrogel has more cells, the presence of vinculin is not as prominent as in the control. These results indicate that the plausible reason why there is a decreased initial number of iMSCs attached to the hydrogels at 24 h of culture could be due to the substrate used to coat the biomaterials, namely, gelatin. For this reason, a different substrate should be evaluated to assess whether the initial cell adherence to the nanocomposite hydrogels could be improved to enable the performance of differentiation studies.

### 3.2. Optimization of iMSC Adhesion on GelMA-BG-MWCNT Nanocomposite Hydrogels

**Figure 3:**
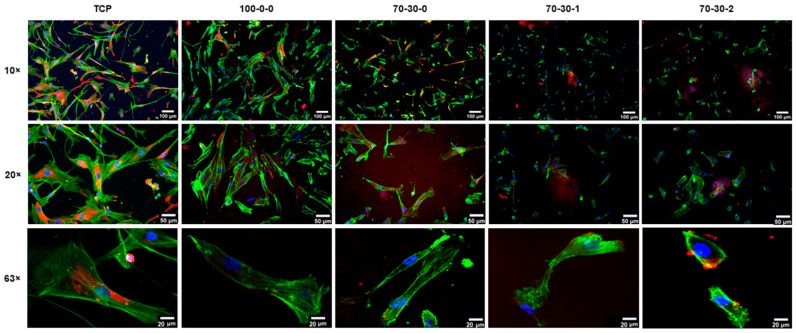
Immunofluorescence microscopy images of iMSCs cultured on fibronectin-coated GelMA-BG-MWCNT nanocomposite hydrogels. Focal adhesions of iMSCs cultured on hydrogels (blue = nuclei; green = F-actin; red = vinculin) at 24 h of culture. A 10× scale bar = 100 µm. A 20× scale bar = 50 µm. A 63× scale bar = 20 µm.

Although gelatin was used to coat TCP surfaces for the maintenance of iMSCs, its application as a coating substrate for nanocomposite hydrogels did not provide an adequate environment for the adhesion of cells. Therefore, a different protein substrate was used to evaluate whether the initial cell adhesion on biomaterials would improve, especially for the nanocomposite hydrogels containing 1 and 2 wt.% MWCNT. Fibronectin, a conserved glycoprotein found in all tissues of the body, was chosen as an alternative cell attachment substrate since it has been previously used for the proliferation and osteogenic differentiation of MSCs [36,42]. Cells cultured on fibronectin-coated hydrogels were stained for vinculin (red), F-actin (green), and nuclei (blue) at 24 h of culture (Figure 3). A considerable increase in initial cell density was observed on the 100-0-0 hydrogel, which appeared similar to that of the control TCP. This indicates that iMSCs cultured on the fibronectin-coated 100-0-0 hydrogel presented a better cell–biomaterial interfacial interaction than the gelatin-coating substrate. This was also observed in the 70-30-0 hydrogel, which not only presented an increased cell adhesion but also an elongated cellular morphology and the formation of a uniform layer of actin filaments.

**Figure 4:**
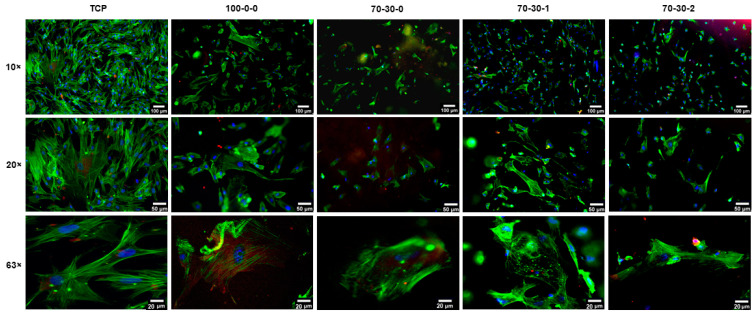
Immunofluorescence microscopy images of pre-differentiated iMSCs cultured on fibronectin-coated GelMA-BG-MWCNT nanocomposite hydrogels. Focal adhesions of iMSCs cultured on hydrogels (blue = nuclei; green = F-actin; red = vinculin) at 24 h of culture. A 10× scale bar = 100 µm. A 20× scale bar = 50 µm. A 63× scale bar = 20 µm.

Cell attachment on the 70-30-1 and 70-30-2 hydrogels was not improved as observed in the 100-0-0 and 70-30-0 hydrogels. However, the expression of vinculin was shown at the periphery of cells cultured on all hydrogels. This observation depicts the presence of integrin-mediated communications between the iMSCs and the surfaces of the hydrogels. Collectively, these results indicate that the initial adherence of iMSCs on biomaterials is better after coating with fibronectin, especially for the 100-0-0 hydrogel, but could be further improved for the 70-30-1 and 70-3-2 nanocomposite hydrogels. Although surface roughness could direct differentiation towards an osteogenic cell lineage [40,43], iMSCs cultured onto the 1 and 2 wt.% MWCNT containing hydrogels could delay this process due to the decreased attachment. The reduced cell adhesion could plausibly be due to the extended period that the iMSCs require to adapt to stiffer surfaces. Bone cells, however, are adapted to proliferate and differentiate in a stiff environment [43]. For this reason, initial cell attachment was further evaluated on pre-differentiated iMSCs to assess whether cell adhesion is enhanced on the 70-30-1 and 70-30-2 hydrogels (Figure 4). Osteogenic differentiation of iMSCs was induced for five days, followed by subsequent seeding onto hydrogels. Figure 4 shows the fluorescent images of pre-differentiated iMSCs cultured on the surfaces of 100-0-0 hydrogel and nanocomposite hydrogels for 24 h. Favorable pre-differentiated cell adhesion was observed on the 100-0-0 and 70-30-0 hydrogels but was improved in the nanocomposite hydrogels containing 1 and 2 wt.% MWCNT (Figure 4) compared to that of the undifferentiated iMSCs (Figure 3). In addition, cells formed an increased layer of elongated actin filaments on the hydrogels, suggesting favorable adhesion and spreading. Based on the results collectively presented in Figure 3 and Figure 4, initial cell adherence on nanocomposite hydrogels was improved and more favorable after coating biomaterials with fibronectin and seeding pre-differentiated iMSCs. The determination of these conditions was crucial to obtain a similar cell density baseline between each biomaterial to further investigate the osteogenic ability of cells cultured on nanocomposite hydrogels. Furthermore, fibronectin functions in several stages of fracture healing by acting as a three-dimensional scaffold immediately following trauma while guiding the assembly of additional ECM components to promote bone regeneration [44]. In addition, it is important to note that the migration of bone cell precursors to the site of injury plays an important role in the healing of bone defects [45,46,47]. Therefore, using fibronectin as a coating substrate and pre-differentiated cells on our nanocomposite hydrogels better mimics the environmental conditions it would encounter for in vivo bone healing applications.

### 3.3. Osteogenic Gene Expression of Differentiated iMSCs Cultured on GelMA-BG-MWCNT Nanocomposite Hydrogels

**Figure 5:**
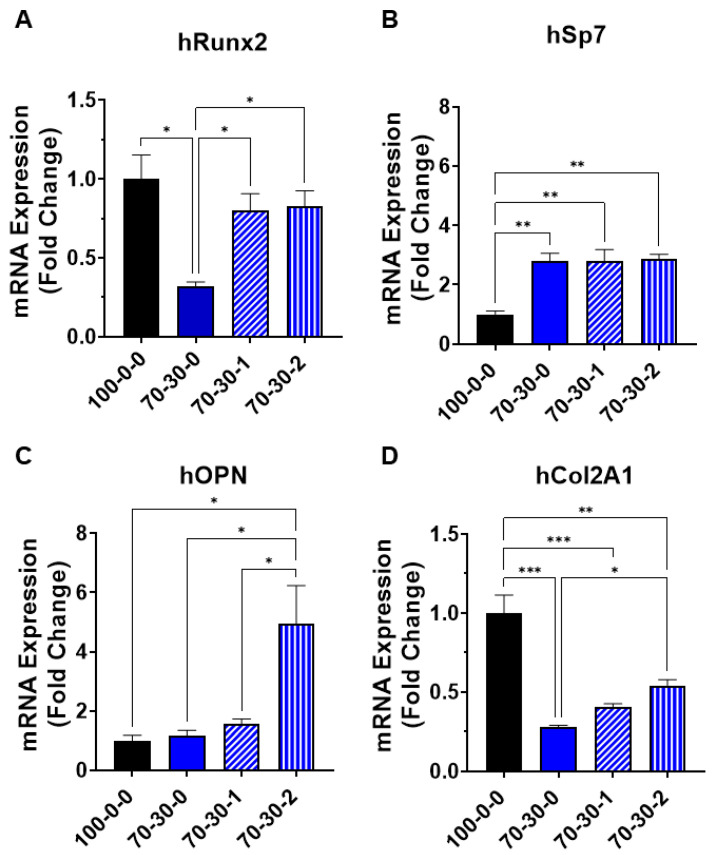
Osteogenic gene expression of differentiated iMSCs cultured on GelMA-BG-MWCNT nanocomposite hydrogels. (**A**) Runx2, (**B**) Sp7, (**C**) OPN, and (**D**) Col2A1 mRNA expressions of pre-differentiated iMSCs cultured on nanocomposite hydrogels that were further differentiated for 15 days. * *p* < 0.05, ** *p* < 0.01, *** *p* < 0.001.

Following the observation that pre-differentiated cells attach better to fibronectin-coated biomaterials, their differentiation potential towards an osteogenic phenotype on GelMA-BG-MWCNT hydrogels was subsequently investigated. Figure 5 shows the osteogenic expression of early and late osteogenic genes in response to differentiation induction on nanocomposite hydrogels. Osteogenic differentiation of iMSCs is a regulated process in which master transcription factors and their target genes control the expression of numerous downstream target genes that code for proteins that determine the osteoblast phenotype, which are necessary for the repair and regeneration of bone [48,49]. There are two master transcription factors that regulate the expression of osteogenic target genes: runt-related transcription factor 2 (Runx2); and transcription factor Sp7, also known as osterix. Runx2 initiates osteogenesis upstream of Sp7 early in the regulatory hierarchy of osteoblast development [50]. Targeted deletion of either genes results in loss of osteoblast differentiation and bone formation [49]. The differentiation of iMSCs cultured on pure GelMA (100-0-0) hydrogels was used as a control to evaluate the effects of BG and MWCNT on the induction of osteogenic differentiation. After 15 days of differentiation, the expression of Runx2 in the 70-30-1 and 70-30-2 nanocomposite hydrogels resulted in a 0.2-fold downregulation with respect to the pure GelMA hydrogel control showing no significance (*p* > 0.05) (Figure 5A). However, Runx2 expression in 100-0-0 hydrogel and nanocomposite hydrogels containing 1 and 2 wt.% MWCNT was significantly higher (*p* < 0.05) than in the 70-30-0 hydrogel. Furthermore, Sp7 gene expression was significantly higher (*p* < 0.05) in the 70-30-0 hydrogel and in the hydrogels containing 1 and 2 wt.% MWCNT, resulting in a 2.8-fold upregulation (Figure 5B). The expressions of Runx2 and Sp7 induce the expression of downstream target genes, such as osteopontin and collagen, which are necessary for mineralization and endochondral ossification for the repair and regeneration of bone [51].

Osteopontin (OPN) presents an RGD motif that binds to integrins and allows the bone cells to adhere to the mineralized matrix [52]. Although OPN has been shown to be an inhibitor of mineralization in a dose-dependent manner by binding to the HCA and inhibiting further growth [53], its presence promotes intrafibrillar mineralization of collagen [54,55]. The major collagenous component of bone ECM is type I collagen. Type 1 collagen has been shown to promote proliferation, survival, adhesion, and osteogenesis in MSCs mediated by α2β1 integrin interaction [56,57,58]. Another form of collagen found in bone is type II. Type II collagen has been linked to the preformation of cartilaginous tissue during the bone fracture healing process [59]. It has been shown that chondrogenic pre-induction of β-TCP/MSC composites presented a significant production of type II collagen and enhanced full bone formation, including marrow organization [60]. Therefore, type II collagen (Col2A1) serves as a modulator during osteogenic differentiation of MSCs via the activation of Runx2 through α2β1 integrin-related FAK signaling pathway and promotes healing of bone defects through an endochondral ossification-like process [59]. Significant upregulation of the OPN gene expression on iMSCs differentiated on 70-30-2 nanocomposite hydrogels (*p* < 0.05) was obtained, resulting in a five-fold upregulation (Figure 5C). Furthermore, the expression of Col2A1 was higher in the 70-30-2 hydrogel compared to the 70-30-0 hydrogel (*p* < 0.05). However, Col2A1 expression significantly decreased in the 70-30-0 (*p* < 0.001) and nanocomposite hydrogels containing 1 (*p* < 0.001) and 2 (*p* < 0.01) wt.% MWCNT, resulting in a 0.73-, 0.6-, and 0.46-fold downregulation, respectively, compared to pure GelMA control (Figure 5D). The smooth surface of 100-0-0 hydrogel could potentially facilitate the increased expression of Col2A1, which is present in cartilage ECM and in the terminal process of endochondral ossification in chondrocyte hypertrophic differentiation [61]. The expression of Col2A1 in nanocomposite hydrogels could plausibly increase if there is a longer period of differentiation. In addition, the incorporation of BG and MWNCT leads to a rougher and stiffer surface in which bone cells, or in this case, pre-differentiated iMSCs, are prone to proliferate, which resulted in the increased expression of early and late osteogenic differentiation markers, as mainly observed in the cases of Sp7 (Figure 5B) and OPN (Figure 5C) genes. The 70-30-1 and 70-30-2 hydrogels presented good early osteogenic expressions (Figure 5A,B) but were mostly enhanced in the 70-30-2 hydrogel for the expression of OPN as a late osteogenic differentiation marker (Figure 5C), which could be due to the possible rough topography of the MWCNT that could further enhance maturation. These results collectively show that GelMA-BG-MWCNT nanocomposite hydrogels possess a favorable environment and ability to further differentiate iMSCs into an osteogenic lineage that could potentially drive bone healing through the process of endochondral ossification.

### 3.4. Osteogenic Protein Expression of Differentiated iMSCs Cultured on GelMA-BG-MWCNT Nanocomposite Hydrogels

**Figure 6:**
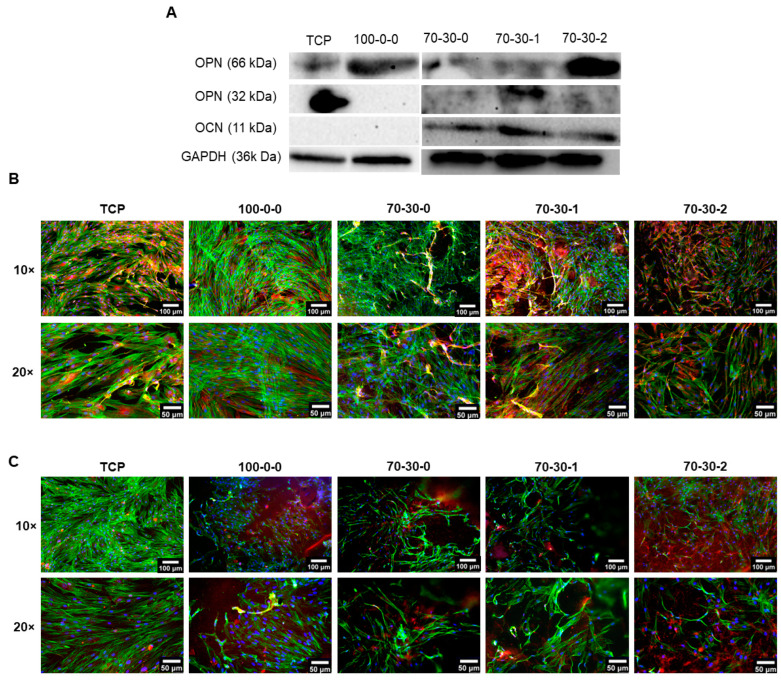
Osteogenic protein expression of differentiated iMSCs cultured on GelMA-BG-MWCNT nanocomposite hydrogels. (**A**) Representative Western blot analysis of the osteogenic proteins OPN and OCN after 15 days of further differentiation of pre-differentiated iMSCs on nanocomposite hydrogels. Immunofluorescence staining of (**B**) osteopontin (OPN) and (**C**) osteocalcin (OCN) in pre-differentiated cells cultured on GelMA-BG-MWCNT nanocomposite hydrogels after 15 days of further differentiation. Cells were stained for nuclei (blue), F-actin (green), and OPN or OCN (red). A 10× scale bar = 100 µm. A 20× scale bar = 50 µm. Please note that in Figure 6A, there are two gels/membranes: one for TCP and 100-0-0 and another for 70-30-0, 70-30-1, and 70-30-2 biomaterials. Because of the different exposures, the consistency of the loading control (GAPDH) should be seen for each membrane separately.

After determining that pre-differentiated iMSCs were able to further differentiate towards an osteogenic phenotype on GelMA-BG-MWCNT hydrogels, Western blot analysis was conducted to investigate the expression of two non-collagenous proteins in differentiated cells cultured on nanocomposite hydrogels for 15 days. One of the characteristic properties used to evaluate the transition towards a mature phenotype is the increase in the expression of bone matrix proteins, OPN and osteocalcin (OCN) [1,62]. The secretion of OPN and OCN should progressively increase as the mineralization process advances for the formation of bone during its repair and regeneration [12,63]. TCP was used as a control. Figure 6A shows that the full-length (60 kDa) OPN protein was detected in all cultures but was mostly expressed in the 70-30-2 hydrogel. OPN is known to be cleaved by matrix metalloproteinases (MMPs) that break down ECM proteins necessary for bone remodeling and regeneration [64,65]. The cleaved 32 kDa OPN fragment protein was also detected and was mostly observed in the 70-30-1 and 70-30-2 hydrogels but was not noticeable in the 100-0-0 hydrogel. In addition, OCN protein was only identified in cells cultured on 70-30-0 hydrogels and nanocomposite hydrogels containing 1 and 2 wt.% MWCNT. Furthermore, immunofluorescence staining was performed on differentiated cells to confirm the presence of OPN (Figure 6B) and OCN (Figure 6C) proteins. OPN protein was most prominently expressed in differentiated iMSCs cultured on nanocomposite hydrogels, specifically on the 70-30-2 hydrogel. In addition, the OCN protein was not as strongly expressed compared to OPN. These results are consistent with the Western blot analysis. Collectively, the effects of BG and MWCNT in nanocomposite hydrogels reveal that these components enhance the expression of mature osteogenic phenotype marker proteins and have the ability to further drive differentiated cells towards a mature osteogenic lineage. The applications of GelMA-BG-MWCNT hydrogels, specifically the 70-30-1 and 70-30-2 hydrogels, could potentially be translated into in vivo models to explore the augmentation of bone regeneration.

### 3.5. Mineralization of Differentiated iMSCs Cultured on GelMA-BG-MWCNT Nanocomposite Hydrogels

**Figure 7:**
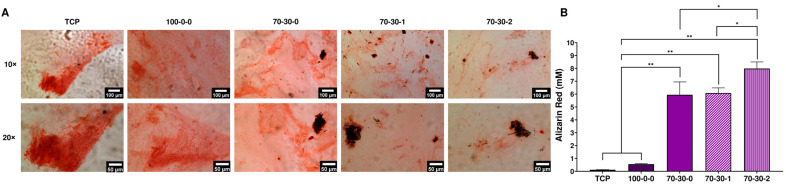
Mineralization of differentiated iMSCs. (**A**) Optical images of Alizarin Red staining of pre-differentiated iMSCs co-cultured with GelMA-BG-MWCNT nanocomposite hydrogels after 15 days of further differentiation. Mineral deposition is stained red. A 10× scale bar = 100 µm. A 20× scale bar = 50 µm. (**B**) Quantification of mineralization by extraction of Alizarin Red from pre-differentiated iMSCs cultured onto the surfaces of GelMA-BG-MWCNT nanocomposite hydrogels after 15 days of further differentiation. * *p* < 0.05, ** *p* < 0.01.

Matrix mineralization is a definitive hallmark of osteoblast differentiation and the last phenotypic stage of osteogenic tissue [12,66]. The bioactive properties of GelMA-BG-MWCNT were previously shown when incubated in simulated body fluid (SBF) [1]. The formation of hydroxycarbonate apatite (HCA) (Ca_10−x_(PO_4_)_6−x_(CO_3_)_x_(OH)_2−x_) layers occurs through the release of soluble ionic species, such as Si, Ca, and P ions, from the BG to form a high-surface-area hydrated silica and polycrystalline HCA bilayer on the glass surface, resulting in an apatite layer similar to that of bone, capable to stimulating the formation of bone tissue [1]. The addition of osteogenic media also provides an exogenous source of phosphate that assists mineralization. Alizarin Red staining was used to visualize and quantify the mineral formation. Since the nanocomposite hydrogels are black due to MWCNT, visualization of the mineralized tissue onto the biomaterials was challenging. Instead, cells were co-cultured with nanocomposite hydrogels to observe the mineral depositions as a function of hydrogel composition. Glass coverslips were placed in TCP before seeding pre-differentiated iMSCs to ease visualization. Figure 7A shows the optical images of mineral depositions of differentiated cells cultured in TCP control and co-cultured with GelMA-BG-MWNCTs after 15 days of further differentiation. A slight increase in stained mineral deposition was depicted in cells co-cultured with 70-30-0 hydrogel and with the nanocomposites containing 1 and 2 wt.% MWCNT, which was observed as dark red to brown spots, evidencing the mineralized nodules. Alizarin staining quantification was performed on pre-differentiated iMSCs cultured on the surfaces of GelMA-BG-MWCNT nanocomposite hydrogels that were further differentiated for 15 days, and their results are shown in Figure 7B. The 70-30-2 hydrogel had the highest mineral deposition after 15 days of further differentiating iMSCs. Mineralization of 70-30-2 hydrogels was significantly higher than 70-30-1 and 70-30-0 (*p* < 0.05) hydrogels and was even more noticeably higher than the 100-0-0 and TCP control (*p* < 0.01). The 70-30-1 and 70-30-0 hydrogels presented similar mineral deposition and were significantly higher than the 100-0-0 and TCP control (*p* < 0.01).

These results show that cells cultured on pure 100-0-0 hydrogel presented a similar mineralization to TCP control after 15 days of further differentiation but could be insufficient to enhance its promotion if pure GelMA were to be used as a bone biomaterial. A significant increase in mineral deposition was, however, observed upon the addition of BG and MWCNT. It could be assumed that the release of soluble ionic species from the BG considerably facilitates the formation of calcium and phosphorous depositions on the surfaces of the nanocomposite hydrogels. In addition, differentiated cells proliferate more on a stiffer and rougher surface that would mimic that of an osseous environment, such as those hydrogels containing BG and MWCNT. This was mostly evident for differentiated iMSCs cultured on 70-30-2 hydrogels, which presented the highest mineralization. The increased mineral deposition in the 70-30-2 hydrogels was also in accordance with the OPN protein expression (Figure 6B), which could plausibly significantly promote the repair and regeneration of bone defects in vivo.

## 4. Conclusions

In this study, GelMA-BG-MWCNT hydrogels were prepared to evaluate the viability and osteogenic ability of iMSCs cultured on the surfaces of nanocomposite hydrogels. iMSCs were chosen for the in vitro studies since they were human-derived and could potentially resemble clinically driven outcomes. Cell interaction studies showed good cytocompatibility properties when culturing iMSCs onto the surfaces of gelatin-coated nanocomposite hydrogels for 7 days. However, the initial cell density was not even amongst cells cultured on hydrogels, and further optimization was required to investigate the osteogenic ability of iMSCs on GelMA-BG-MWCNT hydrogels. Fibronectin substrate was found to promote cell adhesion and, hence, increase the proliferation of cells. This was further enhanced upon seeding pre-differentiated iMSCs for 5 days onto nanocomposite hydrogels. The use of fibronectin coating and pre-differentiated cells on our nanocomposite hydrogels better mimic the environmental conditions it would encounter for in vivo bone healing applications. The promotion of cell adhesion was observed, especially for those biomaterials containing 1 and 2 wt.% MWCNT, possibly due to the rough and stiff surface topography in which pre-differentiated cells are more favorable to mature. Further pre-differentiation of iMSCs on nanocomposites showed that GelMA-BG-MWCNT hydrogels possessed a favorable environment in which the differentiation towards an osteogenic lineage is enhanced. In addition, nanocomposite hydrogels have the ability to promote mature matrix mineralization, which was most evidently observed in differentiated cells cultured on 70-30-2 hydrogels. Although exogenous electrical stimulation was not assessed on cells cultured on nanocomposite hydrogels, it is suggested that its application could further enhance in vitro osteogenic maturation and in vivo bone healing. The obtained results in this study could translate into the potential application of GelMA-BG-MWCNT hydrogels as bone biomaterials that drive the repair and regeneration of bone defects.

## Figures and Tables

**Table 1:** Nomenclature of GelMA-BG-MWCNT nanocomposite hydrogels.

GelMA-BG-MWCNT Nomenclature			GelMA (wt.%)	BG (wt.%)	MWCNT (wt.%)		
100-0-0			100	0	0		
70-30-0	70-30-1	70-30-2	70	30	0	1	2

**Table 2:** Primers for human-specific mRNA amplification.

Gene	Forward (5′ → 3′)	Reverse (5′ → 3′)
Runx2	CCCAGTATGAGAGTAGGTGTCC	GGGTAAGACTGGTCATAGGACC
Sp7	TTCTGCGGCAAGAGGTTCACTC	GTGTTTGCTCAGGTGGTCGCTT
Col2A1	AGCCTGGTGATGATGGTGAA	ACTCTCACCCTTCACACCAG
OPN	TCACCTGTGCCATACCAGTT	TGTGGTCATGGCTTTCGTTG
18S	GCGGTTCTATTTTGTTGGTTT	CTCCGACTTTCGTTCTTGATT

Runx2: Runt-related transcription factor 2; Sp7: Osterix; Col2A1: collagen type II alpha 1 chain; OPN: osteopontin.

## Data Availability

The data that support the findings of this study are available from the corresponding author, Kibret Mequanint, upon reasonable request.

## Supplementary Materials

The following supporting information can be downloaded at https://www.mdpi.com/article/10.3390/biomimetics9060338/s1. Figure S1. Digital image of hydrogels.
