# Supplementary material for: Osteogenic Differentiation Potential of iMSCs on GelMA-BG-MWCNT Nanocomposite Hydrogels
Source: Biomimetics (Basel). 2024 Jun 3;9(6):338. doi: 10.3390/biomimetics9060338 (PMC11201442; doi:10.3390/biomimetics9060338)
Supplement: Supplementary file 1 [file biomimetics-09-00338-s001.zip › biomimetics-2992352-supplementary.pdf]

# Osteogenic Differentiation Potential of iMSCs on GelMA-BG-MWCNT Nanocomposite Hydrogels

**Rebeca Arambula-Maldonado [1] and Kibret Mequanint [1,2,*]**

[1] School of Biomedical Engineering, University of Western Ontario, 1151 Richmond Street, London, ON N6A 5B9, Canada; rarambul@uwo.ca
[2] Department of Chemical and Biochemical Engineering, University of Western Ontario, 1151 Richmond Street, London, ON N6A 5B9, Canada
* Correspondence: kmequani@uwo.ca; Tel.: +1-(519)-661-2111 (ext. 88573)

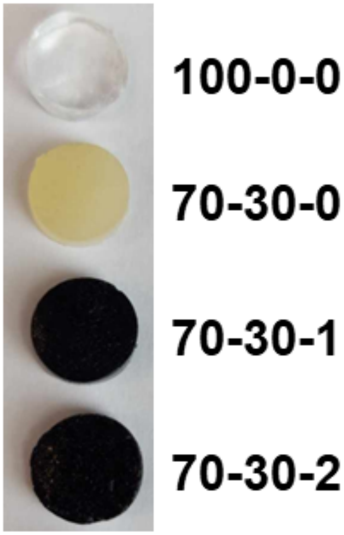


**Figure S1. Digital image of hydrogels.** Image of pure GelMA (100-0-0) hydrogel, 70-30-0, 70-30-1, and 70-30-2 nanocomposite hydrogels.
